# Cardiovascular Responses to a Full Resistance Training Session Performed with and Without Blood Flow Restriction

**DOI:** 10.3390/sports13120430

**Published:** 2025-12-03

**Authors:** Anderson Geremias Macedo, Gabriel de Souza Zanini, Danilo Alexandre Massini, Tiago André Freire Almeida, David Michel de Oliveria, Cátia Caldeira Ferreira, Ricardo Monteiro Robalo, Mário Cunha Espada, Dalton Muller Pessôa Filho

**Affiliations:** 1 School of Sciences (FC), São Paulo State University (UNESP), Bauru 17033-360, SP, Brazil; andersongmacedo@yahoo.com.br (A.G.M.); gabriel.zanini@unesp.br (G.d.S.Z.); dmassini@hotmail.com (D.A.M.); tiagofalmeida.w@gmail.com (T.A.F.A.); 2 PostGraduate Program in Human Development and Technology, São Paulo State University (UNESP), Rio Claro 13506-900, SP, Brazil; 3 Institute of Motricity Sciences, Federal University of Alfenas (UNIFAL), Alfenas 37133-840, MG, Brazil; 4 Santa Clara Campus, Post-Graduate Program in Rehabilitation Sciences, Institute of Motricity Sciences, Federal University of Alfenas, Alfenas 37133-840, MG, Brazil; 5 Post-Graduate Program in Movement Science, School of Sciences (FC), São Paulo State University (UNESP), Bauru 17033-360, SP, Brazil; 6 Department of Physical Education, Faculdades Integradas de Jau (FIJ), Jau 17207-310, SP, Brazil; 7 Centre for Research in Economics and Comparative Development (CIEDEC), Lusíada University of Lisbon, 1349-001 Lisboa, Portugal; 8 Department of Physical Education, Federal University Jataí (UFJ), Jataí 75801-615, GO, Brazil; profdoliveira@ufj.edu.br; 9 Postgraduate Program in Animal Bioscience (PPGBA), Institute of Health Sciences (ICS), Federal University of Jataí (UFJ), Jataí 75801-615, GO, Brazil; 10 Training Optimization and Sports Performance Research Group (GOERD), Faculty of Sport Science, University of Extremadura, 10005 Cáceres, Spain; catia.ferreira@ese.ips.pt; 11 Instituto Politécnico de Setúbal, Escola Superior de Educação (SPRINT, CIEQV), 2914-504 Setúbal, Portugal; ricardo.robalo@ese.ips.pt (R.M.R.); mario.espada@ese.ips.pt (M.C.E.); 12 Centre for the Study of Human Performance (CIPER), Faculdade de Motricidade Humana, Universidade de Lisboa, Cruz Quebrada-Dafundo, 1499-002 Lisboa, Portugal; 13 Comprehensive Health Research Centre (CHRC), Universidade de Évora, 7004-516 Évora, Portugal

**Keywords:** resistance training, blood flow restriction, hemodynamic responses, cardiac response, pressure-rate product

## Abstract

Resistance training (RT) can induce cardiovascular overload, especially at high intensities. Blood flow restriction (BFR) has emerged as a low-load alternative that is potentially effective and safe, although its hemodynamic and respiratory effects remain controversial. Background/Objectives: The aim of this study was to compare cardiovascular responses between a high-load RT session (RT_HL; 70% of one repetition maximum—1RM) and a low-load session combined with BFR (RT_LL+BFR; 30% 1RM). Methods: Nineteen trained men (24.3 ± 3.9 years; 177.7 ± 6.3 cm; 84.7 ± 13.0 kg) performed the RT_HL and RT_LL+BFR protocols, with eight exercises for each protocol. The following variables–heart rate (HR), percentage of maximum heart rate (%HRmax), systolic blood pressure (SBP), diastolic blood pressure (DBP), and rate pressure product (RPP)–were assessed during the protocols. Results: Both protocols significantly increased HR (pre: 74 ± 8 bpm; post: RT_HL = 142 ± 9 bpm; RT_LL+BFR = 133 ± 6 bpm; *p* < 0.01), %HRmax (RT_HL = 72 ± 5%; RT_LL+BFR = 69 ± 4%; *p* < 0.01), SBP (RT_HL = 144 ± 6 mmHg; RT_LL+BFR = 140 ± 6 mmHg; *p* < 0.05), and RPP (RT_HL = 20,469 ± 1620; RT_LL+BFR = 18,637 ± 1253; *p* < 0.01) compared to resting values. No variable exceeded safety thresholds for RPP (<30,000; %HRmax < 75%), and DPB showed a slight elevation in both conditions (*p* < 0.05), but without differences between protocols (*p* = 0.28). Conclusions: Exercise load intensity was an important determinant of hemodynamic responses, but BFR elicited comparable stimuli with low load, thereby emphasizing improved safety to traditional high-load-intensity training.

## 1. Introduction

Resistance training (RT) performed at high loads, typically above 60% of one repetition maximum (1RM), imposes a substantial static component on the musculoskeletal system, which varies according to the intensity and duration of muscular contraction. This component triggers neuromuscular reflexes—mainly the mechanoreflex and metaboreflex—that activate the sympathetic nervous system and elevate cardiovascular demand [1]. As a consequence, there is a marked increase in heart rate (HR) and systolic blood pressure (SBP) [1,2].

During muscular contraction, the mechanical deformation of fibres stimulates mechanoreceptors, while the compression of blood vessels reduces local blood flow, promoting ischemia and the accumulation of metabolites such as lactate, protons, and potassium, which subsequently activate metaboreceptors [3,4,5]. This physiological interaction results in an increase in HR and SBP, which is not always accompanied by proportional adjustments in stroke volume and peripheral vascular resistance, potentially compromising muscle perfusion [6,7,8].

Building on these same mechanisms, the blood flow restriction (BFR) technique aims to potentiate such stimuli by applying external pressure to limb segments through inflatable cuffs, thereby reducing arterial inflow and venous return. Previous studies suggest that RE combined with BFR, even at low loads (20–40% 1RM), can induce cardiovascular responses similar to those observed with high loads, due to increased ischemia and metabolic activation [9,10]. However, the literature remains divergent: while some investigations report greater elevations in SBP and HR during RT with BFR [9], others indicate comparable responses when contrasted with traditional high-load training [8,10].

Although most classical studies have detailed the hemodynamic responses to RT and the use of BFR, in recent decades interest in the topic has grown considerably, particularly due to the technique’s potential in clinical and health-related contexts. Recent reviews and previous statements indicate that BFR, when properly applied, is safe and capable of promoting relevant muscular and cardiovascular adaptations with substantially lower loads [11]. This feature becomes especially important for populations unable to tolerate high training intensities, such as older adults, cardiac patients, and individuals undergoing musculoskeletal rehabilitation. Furthermore, a meta-analysis conducted by Centner et al. [12] demonstrated that RT with low-load intensity and BFR elicits significant improvements in strength and hypertrophy in older adults, comparable to those achieved with high-intensity protocols. Complementarily, Hughes et al. [13] investigated the application of BFR in musculoskeletal rehabilitation and reported consistent benefits in strength and function, without evidence of relevant cardiovascular risks when the method was appropriately monitored.

Most of these studies, however, evaluated only isolated exercises or protocols restricted to a single muscle group, which limits the understanding of hemodynamic responses during complete RT sessions that combine multi-joint and single-joint exercises for both upper and lower limbs. This aspect is particularly relevant, as the total exercise volume and the amount of engaged muscle mass may amplify cardiovascular adjustments during training. Thus, it remains unclear whether, in a full session involving multiple exercises for large muscle groups, the BFR technique elicits cardiovascular responses distinct from those observed in traditional RT_HL. For example, previous studies have reported that multi-joint exercises for the lower limbs elicited higher hemodynamic values than single-joint exercises during both BFR and high-intensity (HI) protocols. Specifically, SBP increased more in the leg press than in the leg extension (+36.1 vs. +16.9 mmHg during high-load (HL) and +47.4 vs. +40.4 mmHg during BFR) [14]. Similarly, the squat exercise (70% 1RM, 6 sets, 15 reps) showed a significantly higher Rate-Pressure Product (RPP), with an increase of 17,019 bpm × mmHg [15], compared to knee extension and flexion exercises (80% 1RM, 3 sets, 10 reps each), where RPP increased by 9845 bpm × mmHg [16]. Hence, load intensity (%1RM), the number of sets, and repetitions have all been shown to influence hemodynamic responses in previous studies that examined the acute responses to a single exercise, whether it involved single- or multi-joint movements. This evidence suggests that the cardiovascular demand should be different during a complete training session. This gap raises a concern regarding the safety of BFR training, as the use of this technique (clinical or practical) has been increasing in fitness centers [17], where training is usually planned with multi-exercise protocols. Therefore, the present study was designed to provide initial insights into the safety of the cardiovascular load associated with BFR (Blood Flow Restriction). To address this necessity, the study compared the hemodynamic responses (such as Rate-Pressure Product (RPP), Systolic Blood Pressure (SBP), and Heart Rate (HR)) elicited by two full resistance training sessions: one involving high load (RE_HL) and the other low load plus BFR (RE_LL+BFR).

In this context, the present study investigated the acute responses to RE_HL (70% 1RM) and RE_LL+BFR (30% 1RM), testing the null hypothesis that load intensity plays a determinant role in modulating the responses, resulting in higher RPP, SBP, and HR responses during RE_HL, whereas BFR would not produce additional significant effects on these variables, despite increased local ischemia.

## 2. Methods

### 2.1. Sample

Nineteen young men, regular practitioners of RE for at least 12 months, participated in the study (age: 24.3 ± 3.9 years; height: 177.7 ± 6.3 cm; body mass: 84.7 ± 13.0 kg). All participants were informed about the study procedures and provided written informed consent. The experimental protocol was approved by the Human Research Ethics Committee of São Paulo State University (UNESP) under CAAE number 19824719.3.0000.5398—FC—UNESP, and was conducted in accordance with the Declaration of Helsinki.

### 2.2. Maximal Strength Test

Dynamic maximal strength was determined using the 1RM test, following the recommendations of Massini et al., [18]. The protocol consisted of: (a) an initial warm-up with light loads and (b) subsequent attempts with progressively heavier loads until concentric failure, defined as the inability to complete more than one full repetition. The rest interval between attempts was 180 s. The test was performed for all exercises included in the training protocols. To minimize localized fatigue, assessments alternated between upper- and lower-limb exercises, involving agonist–antagonist pairs, with a maximum of two exercises per testing session. A 72 h interval was observed between sessions.

### 2.3. Hemodynamic Measurements

Systolic (SBP) and diastolic (DBP) blood pressure were manually measured using a calibrated aneroid sphygmomanometer (BD^®^, São Paulo, SP, Brazil) and a stethoscope (Littmann^®^ Classic III, 3M^®^, St. Paul, MN, USA). All measurements were performed on the left arm, with participants resting in a seated position and the arm supported at heart level, following standardized procedures (i.e., baseline blood pressure was measured twice after a 10 min seated rest, and the mean of both readings was used as the baseline value) [19]. The measurements and monitoring of blood pressure (pre-, during, and post-protocols) were conducted by a single trained assessor with more than 10 years of clinical experience in cardiovascular assessment, thus ensuring methodological consistency and minimizing inter-observer variability.

Blood pressure (BP) records (post-exercise measurements during and after the protocols) were obtained immediately after each exercise bout—within 30–45 s—to capture acute post-exercise hemodynamic responses after each set, thereby avoiding measurement during muscle contraction and the potential interference of intrathoracic pressure changes. It should be noted that this procedure might slightly underestimate the peak systolic pressure during exercise. However, it is considered a reliable and feasible method for obtaining post-exercise information for comparative analysis between resistance training (RT) conditions [20,21]. Indeed, blood pressure measurements taken immediately after exercise have been successfully applied to the analysis of the effect of different load intensities, training volumes, and cuff pressures on hemodynamic responses [22,23].

Heart rate (HR) was continuously monitored throughout all sessions using a chest strap telemetry system (Polar^®^ H10, Kempele, Finland). Maximum heart rate (HRmax) was estimated using the formula HRmax = 220 − age, with an acknowledged individual error margin of approximately 10 bpm [24]. The percentage of maximum heart rate (%HRmax) was calculated for each participant to determine relative cardiovascular intensity. The Rate-Pressure Product (RPP) was calculated as the product HR and SBP. All variables were recorded at three specific time points: rest (5 min before the session), immediately after each exercise bout, and at the end of the training session.

### 2.4. Training Protocol

Participants performed two experimental sessions in a counterbalanced order, separated by at least 72 h: (i) high-load resistance exercise (RE_HL)—intensity of 70% of one-repetition maximum (1RM), three sets of 12 repetitions, with 120 s rest intervals between sets and exercises; (ii) low-load resistance exercise with blood flow restriction (RE_LL+BFR)—intensity of 30% 1RM, three sets of 15 repetitions, with 30 s rest intervals between sets and 180 s between exercises.

Each Resistance Training (RT) session consisted of eight exercises performed sequentially, alternating between upper- and lower-limb movements to engage the main muscles of the whole body: (E1) bench press, (E2) seated row, (E3) triceps extension, (E4) biceps curl, (E5) unilateral knee extension, (E6) leg curl, (E7) leg press, and (E8) standing calf raises. During all RT sessions, participants were continuously monitored for symptoms such as dizziness, nausea, or excessive discomfort. The criteria for exercise termination were defined as (i) systolic blood pressure exceeding 220 mmHg, diastolic exceeding 110 mmHg, reporting any adverse symptoms. However, no participants attended these criteria.

### 2.5. Blood Flow Restriction (BFR)

The BFR was applied using pneumatic cuffs specifically designed for upper and lower limbs (Scientific-arm-WCS and Scientific-leg-WCS, Cardiomed^®^, Curitiba, PR Brazil). For upper-limb exercises, cuffs measuring 80 cm in length and 7 cm in width were positioned bilaterally at the most proximal region of the arms, just below the deltoid insertion and encompassing the upper portion of the biceps brachii. For lower-limb exercises, cuffs measuring 84 cm × 12.5 cm were placed proximally around the thighs, immediately below the inguinal ligament and involving the upper quadriceps region [22].

During the RE_LL+BFR session, cuffs were applied to both arms and both thighs, depending on the limb involved in the exercises. The cuff pressure was set at 50% of resting Systolic Blood Pressure (SBP), which corresponded to 59 ± 3 mmHg for the current sample of participants. The same relative percentage of SBP was applied to both arms and thighs to maintain comparable relative restriction levels across limbs. This pressure value is consistent with a low-to-moderate level and falls within the recommended range based on measurements of Arterial Occlusion Pressure (AOP—i.e., 40–80% AOP, 50 to 100 mmHg).

Cuff pressure was maintained continuously during each set and the rest intervals between sets, but it was reduced to 0 mmHg during the rest intervals between exercises to avoid prolonged ischemia (>10 min) [25]. Prior to the first set of each exercise, the cuffs were re-inflated to the target pressure, and the cuff position and perceived comfort were verified. Pressure was not readjusted during the session, as acute increases in SBP during brief resistance exercise bouts have been shown to be transient and insufficient to compromise the relative occlusion level [26].

The Systolic Blood Pressure (SBP) value was used as the reference instead of Arterial Occlusion Pressure (AOP) determined via Doppler ultrasound, due to practical feasibility and ecological context. Doppler-based procedures require specialized equipment and technical expertise, which limits their applicability in routine training or field-based experimental settings [13]. Moreover, AOP values vary substantially with body position (e.g., sitting, standing, or supine), which adds challenges when performing multiple exercises for the upper and lower limbs within the same session [27].

### 2.6. Rating of Perceived Exertion (RPE)

The RPE scores were obtained using the Borg CR-10 scale (ranging from 0 (no effort) to 10 (maximal effort)). After the three sets of each exercise, and with the cuff still inflated, participants verbally indicated the number corresponding to the exertion level just performed. Participants were first familiarized with the scale before data collection and asked to respond to the question: “How intense was your effort during this exercise?” The average RPE for the entire training protocol (RPE-P) was calculated as the mean of the scores verbally indicated by the participants after each exercise [22].

### 2.7. Statistical Analysis

Data normality was assessed using the Shapiro–Wilk test. To compare training sessions (RE_HL and RE_LL+BFR) and time points (rest, post-exercise, and recovery), a two-way analysis of variance (ANOVA) was applied, followed by Tukey’s post hoc test when appropriate. Effect sizes were estimated using partial eta squared (η^2^p) and interpreted as small (0.0099), medium (0.0588), or large (0.1379) [28]. Associations between hemodynamic responses (HR, percentage of maximum heart rate—%HRmax, SBP, and RPP) at the end of each session were evaluated using Pearson’s correlation coefficient, adjusted for age. Correlation magnitudes were classified as negligible (<0.29), low (0.30–0.49), moderate (0.50–0.69), high (0.70–0.89), or very high (0.90–1.00). Statistical power SP was calculated using G*Power (3.1) for the main comparisons of hemodynamic responses (SBP, DBP, HR, and RPP) between protocols, as well as for the correlation coefficients between these responses. The sample power (SP) analyses were determined by considering a 95% confidence level (alpha = 0.05, representing a two-tailed test), the observed pre- vs. post-protocol values for hemodynamic variables, the effect size (partial eta squared (η^2^p), and the Pearson correlation coefficient (r). For all analyses, the level of significance was set at *p* ≤ 0.05.

## 3. Results

The HR increased significantly in both conditions compared with rest (F_[1,36]_ = 22.6, *p* < 0.01, η^2^p = 0.39 large, SP = 100%). At the post-session time point, HR was higher in RT_HL compared with RT_LL+BFR (*p* < 0.01; SP = 82%), while resting values did not differ between protocols (*p* = 0.29). Similar results were observed for %HRmax (RT_HL vs. RT_LL+BFR; t_[36]_ = 2.809, *p* < 0.01; SP = 78%), confirming a consistent increase after exercise regardless of RT protocol (Table 1).

SBP and RPP also increased significantly in both sessions (SBP F_[1,36]_ = 307.3, *p* < 0.01, η^2^p = 0.89 large, SP = 100%; RPP F_[1,36]_ = 196.7, *p* < 0.01, η^2^p = 0.84 large, SP = 100%). At the post-session time point, RT_HL presented higher values of SBP (*p* = 0.03; SP = 70%) and RPP (*p* < 0.01; SP = 88%), although no differences were observed at rest (Table 1). Diastolic blood pressure (DBP) showed a slight elevation in both conditions (*p* < 0.05), but without differences between protocols (*p* = 0.28, g = 0.18 insignificant, SP = 15%).

**Figure 1:**
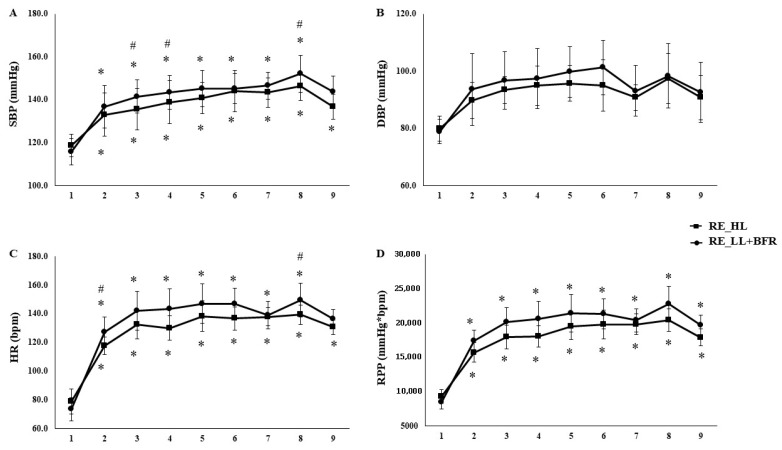
Comparison of cardiovascular responses (SBP, DBP, HR and RPP) across individual exercises in high-load resistance exercise (RT_HL) and low-load resistance exercise with blood flow restriction (RT_LL+BFR). Systolic blood pressure (SBP—Panel (**A**)), diastolic blood pressure (DBP—Panel (**B**)), Heart rate (HR—Panel (**C**)), and rate-pressure product (RPP—Panel (**D**)) measured at rest (1) and post each exercise: (2) bench press, (3) seated row, (4) triceps extension, (5) biceps curl, (6) unilateral knee extension, (7) leg curl, (8) leg press, and (9) standing calf raises in high-load resistance exercise (RT_HL) and low-load resistance exercise with blood flow restriction (RT_LL+BFR). Data are expressed as mean ± SD. * Significant difference from rest (*p* < 0.05). # Significant difference between protocols at the same exercise (*p* < 0.05).

Analysis by individual exercises revealed that RT_HL produced higher values of HR (F_[5.3,191.2]_ = 5.763, *p* < 0.01, η^2^p = 0.138 large, SP = 99%), %HRmax (F_[5.4,192.2]_ = 5.345, *p* < 0.01, η^2^p = 0.129 medium, SP = 99%), and RPP (F_[5.8,208.7]_ = 5.673, *p* < 0.01, η^2^p = 0.136 medium, SP = 99%) in almost all exercises (E1–E7; *p* < 0.01), with convergence between protocols only in the final exercise (E8). SBP was greater in RT_HL at specific points (E2, E3, and E7; *p* < 0.01) (Figure 1). During the recovery phase, HR remained significantly elevated compared with rest in both sessions (*p* < 0.01), but was higher following RT_HL (HR: *p* = 0.02; %HRmax: *p* < 0.01) (Figure 1).

Correlation analyses indicated moderate-to-high associations between the responses of the two protocols for HR (r = 0.46; *p* = 0.02, SP = 51%), %HRmax (r = 0.47; *p* = 0.03, SP = 53%), SBP (r = 0.41; *p* = 0.03, SP = 41%), and RPP (r = 0.40; *p* = 0.04, SP = 40%), suggesting similar response patterns across conditions.

The RT protocols elicited moderate to high scores of RPE. Mean RPE-P values were 7.9 ± 0.6 for RT_HL and 5.7 ± 0.7 for RT_LL+BFR, with both ratings high correlated (r = 0.63, *p* < 0.01, SP = 84%). The RT_HL produced significatively higher scores than RT_LL+BFR (*p* > 0.05), suggesting that the BFR protocol was well tolerated, and can be performed with lower level of perceptual exertion than RT_HL.

## 4. Discussion

The present study compared cardiovascular responses during RT sessions performed with high load and with low load combined with BFR. Our findings demonstrated that both protocols elicited significant increases in HR, %HRmax, SBP, and RPP compared with rest; however, the high-load condition (RT_HL) produced more pronounced hemodynamic responses at several points during the session, particularly between the initial and intermediate exercises. Thus, our hypothesis that load intensity would be the primary determinant of cardiovascular responses was confirmed.

The observed peak RPP and %HRmax values remained within commonly accepted moderate safety ranges (RPP < 30,000 bpm × mmHg; %HRmax < 75%) for healthy, resistance-trained individuals [18,29,30,31]. These results support the acute safety of BFR when properly supervised. The results for HR and RPP corroborate previous investigations reporting greater cardiac workload during high-intensity RE, largely attributed to increased motor unit recruitment, the magnitude of the static component, and stimulation of muscular mechanoreceptors [2,8]. Particularly, the static component of resistance exercise (i.e., sustained muscular tension that compresses intramuscular vessels) is of great influence, since it increases peripheral resistance and stimulating mechanoreceptors, consequently elevating sympathetic output and hence blood pressure [23,29,30]. Although BFR is theorized to augment metaboreceptor activation due to greater intramuscular metabolite accumulation [31], our data did not demonstrate additional hemodynamic stress beyond that observed with traditional RE. Similar findings were reported by Vilaça-Alves et al. [16], who observed no significant differences in SBP or HR between high-load protocols and low-load BFR exercise.

Despite the current findings are suggesting that the demand on cardiac and circulatory system is reduced in young male adults, the low risk might be clinically important for population with chronic diseases such as hypertension or cardiopathy, to whom abrupt elevations in SBP and RPP may represent increased risk. Previous study has been evidenced that patients with coronary artery disease who performed resistance training with BFR, improved muscular strength and hemodynamic parameters without impairments in vascular function, supporting the safety and clinical applicability of the method when appropriately monitored [32]. In addition, other studies involving hypertensive individuals suggested that BFR training can reduce resting and exercise blood pressure following supervised interventions [33,34]. Indeed, low-load RT with BFR has been shown to be more effective in reducing SBP and improving autonomic function than traditional high- or low-intensity RT without BFR in hypertensive patients [34]. Nevertheless, the current RT session using the BFR protocol should be viewed with caution when recommending it to clinical populations, as their potential risks differ from those in healthy individuals [35]. However, the current findings corroborate the notion that low occlusion pressures and appropriate cuff dimensions are recommended to reduce the strain on the cardiac and respiratory systems [25,35].

Moreover, the hemodynamic responses observed after each exercise in the current study are aligned with recent findings. For example, recent meta-analysis involving 160 participants (79% men and 21% women), aged between 18 and 67, and RT planned with load intensity between 20 and 30% 1RM for BFR and between 70 and 80% 1RM for HI. These studies, comprising one to four exercises, three to six sets per exercise, and repetitions ranging from 10 to 30 for BFR and 8 to 15 for HI, showed no significant difference between BFR and HI for heart rate and systolic blood pressure, despite rate pressure product differing between protocols [7].

Therefore, the reports in literature support the assumption that BFR intensities of 20% to 30% of 1RM or produced similar HR and SBP responses at the end of the session—as well as SBP and DBP up to 60 min post-exercise—to those produced by 70% to 80% of 1RM protocols, despite the lower RPP after BFR exercise [10,14,16]. Hemodynamic responses have been reported after the execution of either a single isolated exercise [10,14,15] or multiple exercises performed together or across different body segments [9] during LL+BFR and HI protocols. Collectively, these studies have shown that hemodynamic responses were higher for multi-joint exercises than single-joint exercises in both BFR and HI protocols [18]. These findings further support the current results by demonstrating that LL+BFR training session elicited hemodynamic responses comparable to a traditional high-load training session, but with reduced cardiovascular strain.

Other studies investigating periodized and individualized programs combining resistance and endurance training have reported chronic reductions in resting SBP (from 126.9 ± 9.7 to 120.5 ± 8.8 mmHg, *p* = 0.013) after six weeks, despite observing no changes in resting HR or DBP [36]. Similarly, BFR did not enhance physiological function during periods of reduced load or recovery, with no significant changes in resting heart rate (HR) or blood pressure (BP) observed during a high-frequency training transition phase [37]. Specifically, the stability of hemodynamic function—rather than reduction—might be a favourable stimulus of RT with LL+BFR when volume-load is reduced or recovery is prioritized. However, the potential for a beneficial effect (such as supercompensation) while minimizing mechanical stress remains unaddressed.

Another relevant finding is the demonstration of minimal differences between conditions, with the responses not exceeding established safety thresholds for cardiovascular demand. In both protocols, the RPP and %HRmax reached values recommended by international exercise guidelines [19,38,39]. These results, therefore, reinforce the notion that young, healthy individuals can safely perform both RT_HL and RT_LL+BFR for improving muscle force, conditioning, and mass.

In addition to the hemodynamic findings, the perceptual data provided valuable insight into the tolerability of the RT protocols. Both conditions elicited moderate-to-high rates of perceived exertion, with differences suggesting that RT sessions planned with high-load intensity was perceptually a distinguishable effort from RT planned with low-load intensity [22]. Despite the fact that discomfort during an unusual training condition (e.g., low load) in combination with cuff pressure might be factors contributing to the increase in RPE response [40], the current study is aligned with the assumption that resistance exercise is perceived as harder when performed with high-intensity loads and multiple sets involving small and large muscles [22]. Indeed, the high mechanical stress during RT_HL is linked to the stimulation of fast-twitch fiber recruitment. This process requires large motor units firing at high rates, and has a strong influence on the sensory and motor cortex modulation of conscious perception of exertion [41], Therefore, an RT session with BFR is not only a safety exercise in terms of hemodynamic responses, but also more comfortable alternative for populations where high mechanical stress is contraindicated.

From a physiological perspective, BFR emerges as an effective alternative for individuals who require optimization of hypertrophic and strength adaptations without high mechanical loads. The technique has been shown to enhance muscle activation and acutely stimulate molecular signalling pathways linked to hypertrophy, including greater phosphorylation of key proteins and increased expression of ribosomal transcription factors—adaptations comparable to those observed with traditional high-load protocols [42,43,44]. Moreover, BFR training involves a lower risk of orthopaedic injury and reduced cardiovascular strain, which may be particularly beneficial for athletes in rehabilitation and populations unable to tolerate high loads [43,44,45].

Indeed, previous studies have supported the interpretation that different combinations of volume and intensity during RT are strong modulators of cardiovascular acute responses. For example, Gjovaag et al. [46] demonstrated that higher loads are associated with greater metabolite production and autonomic activation, resulting in more pronounced increases in HR and SBP, regardless of the presence of localized ischemia. In line with this, Libardi et al. [47] observed that RE performed to failure, with or without BFR, elicited comparable hemodynamic responses, suggesting that contractile and metabolic stress, rather than the use of BFR itself, are the primary determinants of cardiovascular load.

Although the present study did not directly assess blood lactate concentration, it is plausible that metabolic mechanisms associated with BFR contributed to the responses observed. Evidence indicates that RT_LL+BFR promotes greater accumulation of metabolites such as lactate and H^+^ ions, even at 20–40% 1RM, thereby stimulating metaboreceptors and enhancing autonomic responses [48,49,50]. Studies have been showed that RT with BFR elevates blood lactate concentration to approximately 6–8 mmol·L^−1^ at 30 to 40% 1RM. This range of values has also been observed during RT with traditional high-load intensity (e.g., 70 to 85% 1RM) [22,48,51]. Therefore, these reports support the notion that metabolic stress (i.e., metabolic acidosis due to the increased glycolysis activation induced by ischemia) might be an important stimulus for autonomic activation during RT with BFR, despite the lower mechanical load. Nonetheless, current results demonstrate that RT_HL elicited higher hemodynamic responses than low-load BFR, reinforcing the concept that contractile intensity and the total muscle mass recruited are the main drivers of increases in HR, SBP, and RPP.

This study, however, has limitations that should be acknowledged. First, the sample size was limited to young, physically active men. Although these findings suggest that BFR may represent a lower-load strategy with reduced cardiovascular stress, this interpretation applies strictly to healthy, resistance-trained young men. Future studies including older adults and clinical populations are necessary to confirm whether similar hemodynamic patterns occur in these groups. Second, blood pressure was measured manually. This approach might introduce variability due to the lower temporal resolution and greater susceptibility to human error when compared to automated and continuous non-invasive systems, despite all measurements being performed by a single experienced evaluator to minimize observer bias. Future investigations employing beat-to-beat or continuous blood pressure monitoring (e.g., Finapres^®^, CNAP^®^, Enschede, the Netherlands) could provide more accurate temporal profiles of acute cardiovascular responses during and immediately after resistance exercise. Third, no direct measurements of cardiac function (such as an electrocardiogram) were assessed, thereby limiting detailed information on cardiac control during the BFR protocol. Finally, the use of %SBP instead of %AOP during training is a limitation, as the effects of the BFR can be underestimated with the actual pressure. Moreover, given the theoretical range for OAP (100–210 mmHg) [35], the 50% SBP likely falls within the recommended zone of cuff pressures (40–80% AOP) which is considered ideal for optimize BFR effects and reducing risks [25].

## 5. Conclusions

Both high-load (HL) and low-load with blood flow restriction (LL+BFR) resistance training sessions elicited significant increases in HR, SBP, and RPP compared with rest, with higher responses observed during the high-load session. These findings confirm that load intensity is an important determinant of cardiovascular demand during resistance exercise, while BFR does not impose additional systemic burden. Perceptual data indicated similar effort between RT sessions, suggesting that RT with low-load and BFR was well tolerated. Collectively, these results support RT with BFR as an effective and safe strategy to induce physiological stress with lower mechanical load. This represents a promising training alterative for healthy individuals with impairments (transitory or permanent) that prevent them from lifting heavy loads, and an appropriate protocol to be further investigated regarding safety in populations requiring special care, such as older adults, hypertensive individuals, and cardiac patients.

## Figures and Tables

**Table 1:** Mean values ± SD of hemodynamic variables at rest and post-session during RT_HL and RT_LL+BFR.

Variables	RT_HL			RT_LL+BFR			Protocols Differences
	Rest	Post	*p*	Rest	Post	*p*	*p*
**HR** **(bpm)**	74 ± 8	142 ± 9	<0.01 *	78 ± 8	133 ± 6	<0.01 *	<0.01 **
**%HRmax (%)**	39 ± 5	71 ± 5	<0.01 *	40 ± 4	69 ± 4	<0.01 *	0.03 **
**SBP** **(mmHg)**	119 ± 2	144 ± 6	<0.01 *	119 ± 5	140 ± 6	<0.01 *	<0.05 **
**DBP** **(mmHg)**	79 ± 4	97 ± 7	0.04 *	80 ± 4	93 ± 5	<0.05 *	0.28
**RPP**	8513 ± 975	20,469 ± 1620	<0.01 *	9257 ± 907	18,637 ± 1253	<0.01 *	<0.01 **

* Significant difference from rest values; ** Significant difference between RT_HL and RT_LL+BFR; Post: post exercise; HR: heart rate; %HRmax: percentage of maximum heart rate; SBP: systolic blood pressure; DBP: diastolic blood pressure; RPP: rate-pressure product.

## Data Availability

The data that support the findings of this study are available from the last author (dalton.pessoa-filho@unesp.br) upon reasonable request.

## Abbreviations

The following abbreviations are used in this manuscript:

RE	Resistance Exercise
BFR	Blood Flow Restriction
RE_HL	High-Load Resistance Exercise
RE_LL+BFR	Low-Load Resistance Exercise combined with Blood Flow Restriction
1RM	One Repetition Maximum
HR	Heart Rate
%HRmax	Percentage of Maximum Heart Rate
SBP	Systolic Blood Pressure
DBP	Diastolic Blood Pressure
RPP	Rate Pressure Product
η^2^p	Partial Eta Squared
g	Hedges’ g
SD	Standard Deviation
